# Supplementary material for: CTCF orchestrates the germinal centre transcriptional program and prevents premature plasma cell differentiation
Source: Nat Commun. 2017 Jul 5;8:16067. doi: 10.1038/ncomms16067 (PMC5504274; doi:10.1038/ncomms16067)
Supplement: Supplementary Information [file ncomms16067-s1.pdf]

Title of file for HTML: Supplementary Information
Description: Supplementary Figures and Table.

Title of file for HTML: Supplementary Data 1
Description: List of genes differentially expressed between CTCFfl/fl and CTCFfl/+ hCD2+ sorted B cells stimulated for 48h in the presence of antiCD3/antiCD28 and T cells

Title of file for HTML: Supplementary Data 2
Description: List of genes differentially expressed between CTCFfl/fl and CTCFfl/+ hCD2+ sorted B cells stimulated for 48h in the presence of LPS/IL4

Title of file for HTML: Peer Review File
Description:

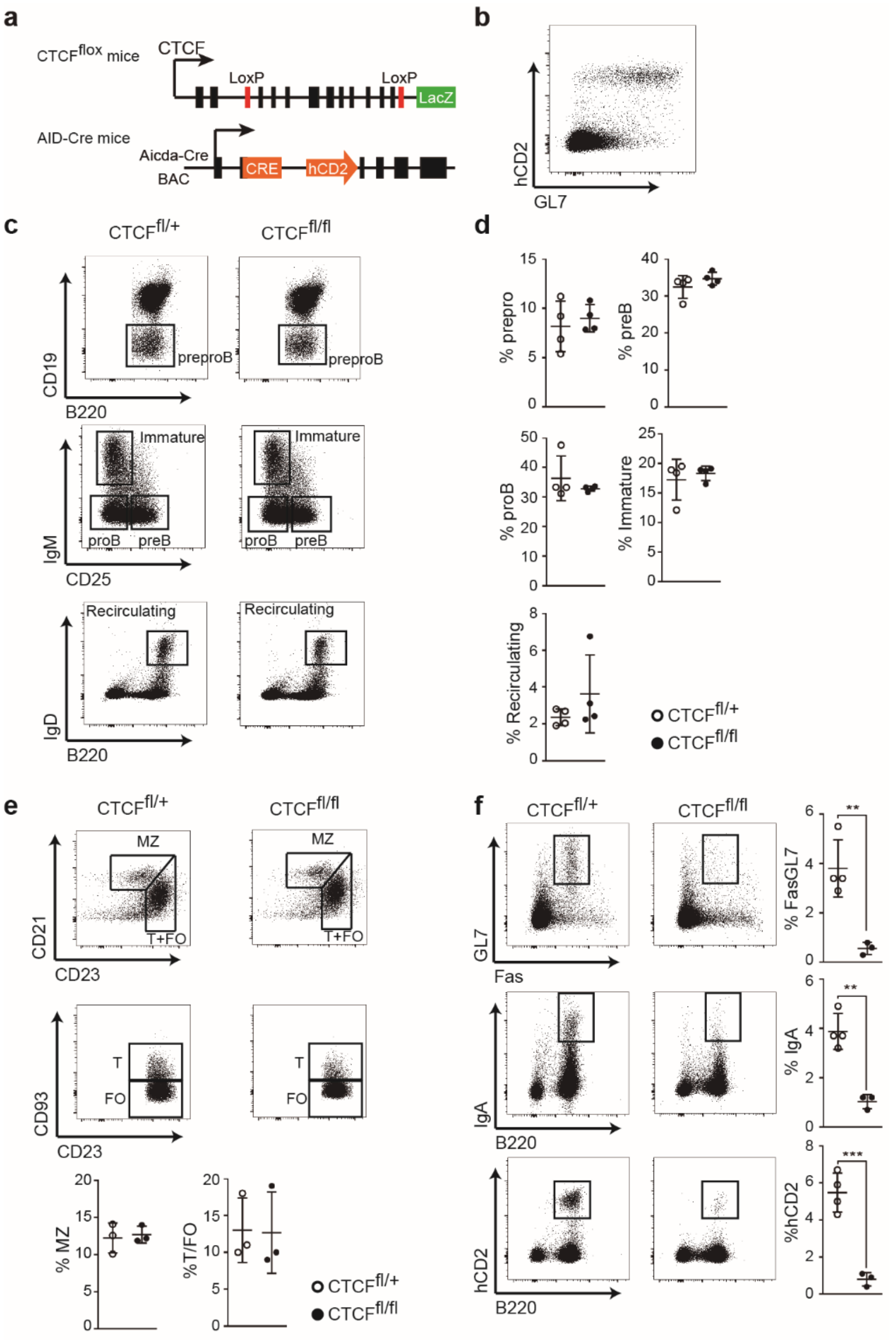

Supplementary Figure 1. B cell differentiation and constitutive GC in CTCF deficient mice. A) Representation of the constructs used for conditional depletion of CTCF in GC B cells. Mice carrying the CTCF allele flanked by LoxP sites (top) were bred to transgenic AID-CRE mice (bottom), where a cassette encoding for Cre recombinase and the hCD2 receptor is inserted in an AID-BAC. B) FACS analysis of hCD2 expression in Peyer's Patch B cells from CTCF$^{fl/+}$ mice. C) FACS analysis of B cell differentiation in bone marrow. Populations were defined as follows: preproB (B220^{+} CD19^{-}); proB (B220^{+}CD19^{+}CD25^{-}IgM^{-}); preB (B220^{+}CD19^{+}CD25^{+}IgM^{-}); Immature (B220^{+}CD19^{+}CD25^{-}IgM^{+}); Recirculating (B220^{+}CD19^{+}IgD^{+}). D) Quantification of the bone marrow populations shown in (C). Absolute numbers are shown in Supplementary Table 1. E) FACS analysis of B cell differentiation in spleen. B cell populations were defined as follows: Transitional (T: B220^{+}CD21^{+}CD23^{++}CD93^{-}); Marginal Zone (MZ: B220^{+}CD21^{++} CD23^{+}); Follicular (FO: B220^{+}CD21^{+}CD23^{++}CD93^{-}). Quantifications are shown on the right. Absolute numbers are shown in Supplementary Table 1. All population percentages are referred to total B220^{+}. CTCF$^{fl/+}$, white dots; CTCF$^{fl/fl}$; black dots. F) FACS analysis of GL7, Fas, IgA and hCD2 expression in Peyer's Patches B cells from CTCFfl/+ (n=4) and CTCFfl/fl (n=3) mice. Plots are gated on B220+ cells (top) or on total live lymphocytes (middle and bottom). Quantifications are shown on the right, as percentages within B220+ cells. p(FasGL7)= 0.0056; p(IgA)= 0.0015; p(hCD2)= 0.0008. Statistical analysis was done by two-tailed unpaired Student's *t* test.

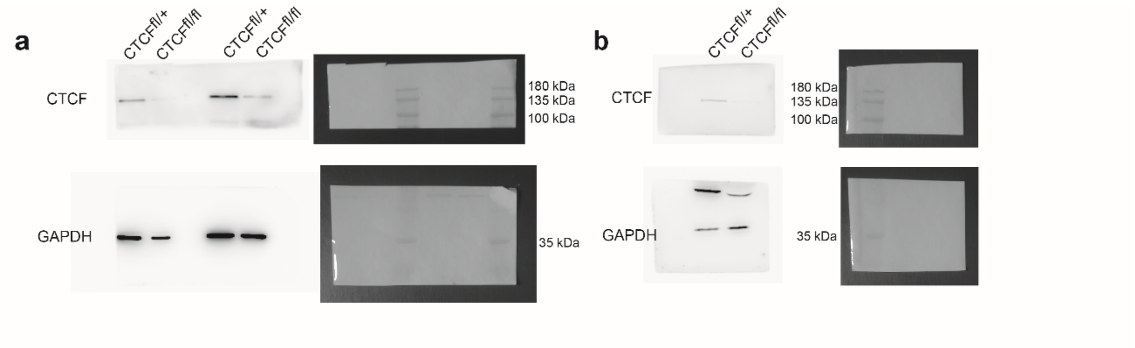

Supplementary Figure 2. Uncropped western blots. A) Western blot analysis of CTCF and GAPDH in isolated hCD2$^+$ B cells from CTCF$^{fl/+}$ and CTCF$^{fl/fl}$ mice after 72 hours of LPS/IL4 stimulation. B) Western blot analysis of CTCF and GAPDH in isolated hCD2$^+$ B cells from CTCF$^{fl/+}$ and CTCF$^{fl/fl}$ mice after 72 hours of stimulation in CD3/CD28 T-B co-cultures.

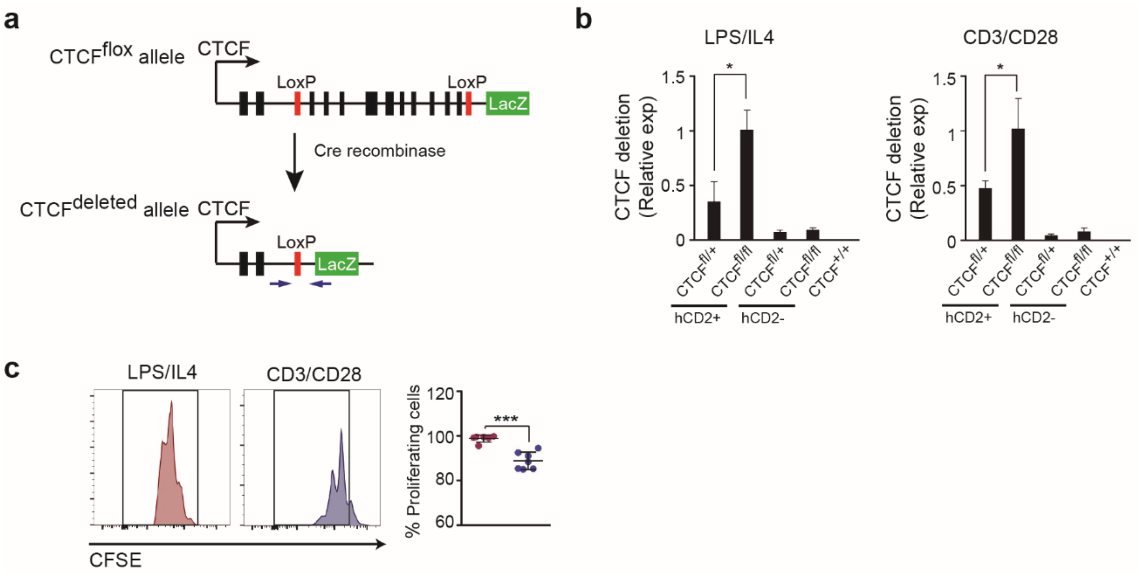

Supplementary Figure 3. Analysis of CTCF deletion and comparison of proliferation between LPS/IL4 and CD3/CD28 stimulated B cells. A) Representation of the strategy used for detection of CTCFfl allele deletion by qPCR. Upper, CTCF^{fl} allele before Cre-mediated recombination. Lower, CTCF^{fl} allele after Cre-mediated recombination. Blue arrows represent primers used in the PCR. B) qPCR analysis of CTCF deletion in hCD2^{+} and hCD2^{-} cells from CTCF$^{fl/+}$ (n=3) and CTCF$^{fl/fl}$ (n=3) mice after 48h of stimulation with LPS/IL4 (right panel) or CD3/CD28 and T cells (left panel). p(LPS/IL4)= 0,0112; p(CD3/CD28)= 0,0283. C) CFSE staining of hCD2^{+} spleen B cells from CTCF$^{fl/+}$ (n=5) and CTCF$^{fl/fl}$ (n=7) mice after 72h of stimulation with LPS/IL4 or CD3/CD28 and T cells. Quantifications are shown on the right as percentages within B220+CD2+ cells. p<0.0001. Numbers indicate percentages ± Standard Deviation. Statistical analysis was done by two-tailed unpaired Student's *t* test.

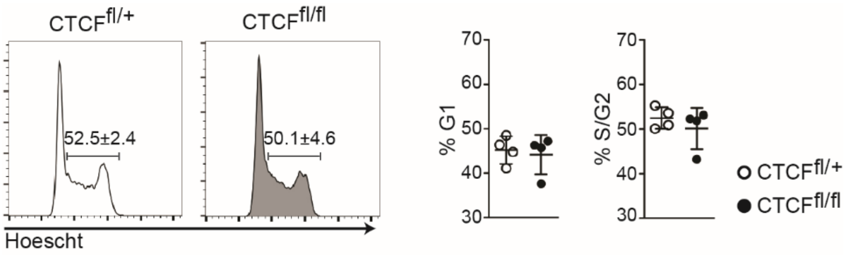

Supplementary Figure 4. RNA-seq analysis of LPS/IL4 and CD3/CD28 stimulated B cells. A) Gating strategy and post-sort analysis for selection of B cells used in RNA-seq experiments. CTCF$^{fl/+}$ and CTCF$^{fl/fl}$ B cells stimulated for 48h hours either in the presence of LPS/IL4 or in the presence of CD3/CD28 T stimulation were sorted as DAPI-, B220+, hCD2+ cells. Representative pre- and post-sort plots of B cells stimulated with CD3/CD28 T cells are shown. B) Heatmap analysis of RNA-seq data of cell cycle-related genes. Cell cycle genes (484) were chosen using GO Enrichment Analysis. Z-scores for resting, GC, LPS/IL4 and CD3/D28 stimulated B cells are represented. Clustering was performed using the average linkage method based on Pearson correlation distance. Each column shows an independent replicate.

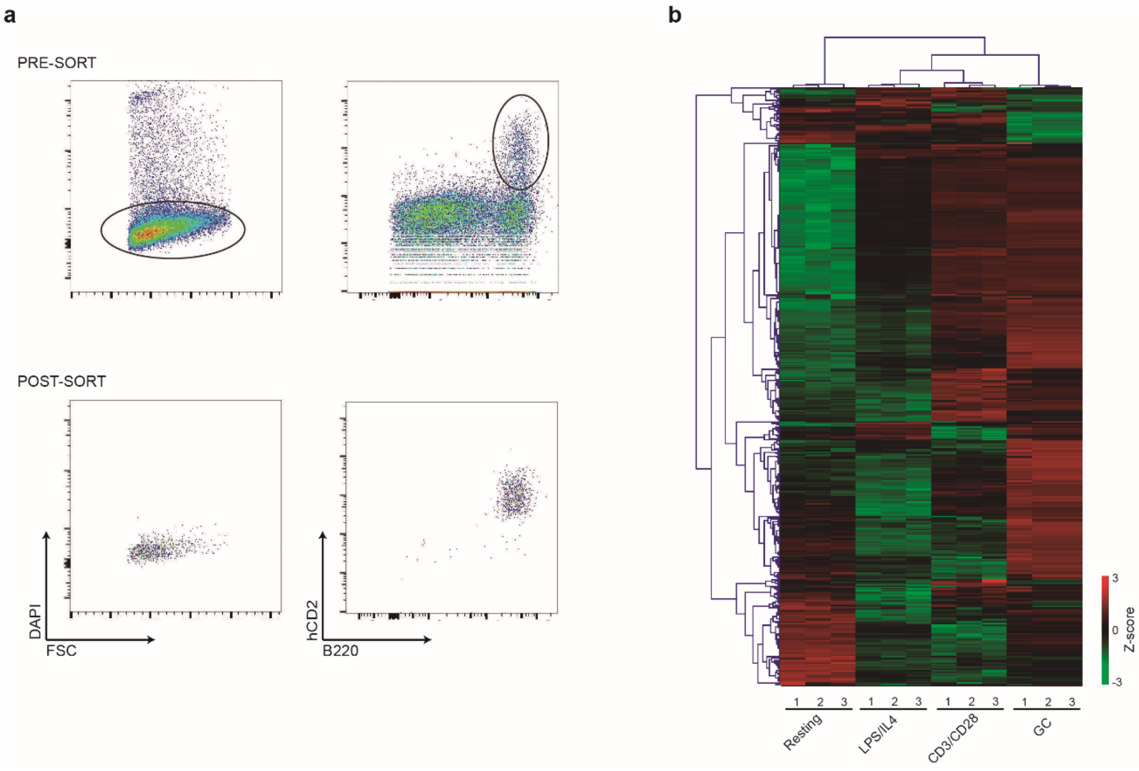

Supplementary Figure 5. Cell cycle analysis in LPS/IL4 stimulated B cells. FACS cell cycle analysis of hCD2^{+} cells from CTCF$^{fl/+}$ (n=4) and CTCF$^{fl/fl}$ (n=4) mice after 48h of stimulation with LPS/IL4. Numbers indicate percentages ± Standard Deviation. Quantification of G1 and S/G2 phase proportions is shown on the right.

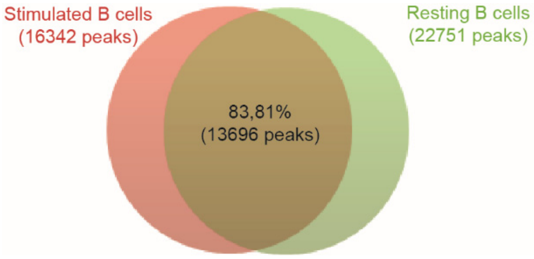

Supplementary Figure 6. Comparison of CTCF binding in LPS/IL4 stimulated and naïve B cells. ChIP-seq analysis of CTCF binding peaks in stimulated B cells and resting B cells from GSE43594[1]. Overlap of common peaks are depicted as Venn diagram.

		Cell number	
**Subset**	**Tissue**	**CTCF$^{fl/+}$**	**CTCF$^{fl/fl}$**
**PreproB**	Bone marrow[a]	0,95±0,20	1,24±0,13
**ProB**	Bone marrow[a]	3,78±0,39	4,12±0,19
**PreB**	Bone marrow[a]	3,60±0,68	4,36±0,28
**Immature**	Bone marrow[a]	1,96±0,42	2,28±0,33
**Recirculating**	Bone marrow[a]	0,70±0,11	1,37±0,51
**Mature**	Spleen[b]	3,48±1,38	3,72±1,39
**Follicular**	Spleen[b]	1,91±0,70	2,14±0,82
**Transitional**	Spleen[b]	0,23±0,06	0,27±0,10
**Marginal zone**	Spleen[b]	0,45±0,21	0,45±0,16

a: cell number x10^5±SEM
b: cell number x10^7±SEM

Supplementary Table 1. Absolute cell number of bone marrow and splenic B cell subsets in CTCF$^{fl/+}$ and CTCF$^{fl/fl}$ mice.

SUPPLEMENTARY REFERENCE

1. Thomas-Claudepierre AS, Schiavo E, Heyer V, Fournier M, Page A, Robert I*, et al.* The cohesin complex regulates immunoglobulin class switch recombination. *The Journal of experimental medicine* 2013, **210**(12)**:** 2495-2502.
